# Knowledge of obstetric danger signs and its associated factors among husbands residing in the Raya Kobo district, Ethiopia

**DOI:** 10.3389/fpubh.2024.1466218

**Published:** 2024-12-31

**Authors:** Jemal Derbew, Adem Yesuf, Abebe Mihrete, Amira Abdallah, Abdurehman Mohammed, Nurye Sirage, Bogale Molla

**Affiliations:** ^1^Bati Primary Hospital, Bati, Ethiopia; ^2^School of Midwifery, College of Health Sciences, Woldia University, Woldia, Ethiopia; ^3^Department of Nursing, College of Medicine and Health Sciences, Debre Berhan University, Debre Berhan, Ethiopia; ^4^Department of Nursing, College of Medicine and Health Sciences, Worabe University, Worabe, Ethiopia; ^5^Department of Public Health, College of Medicine and Health Sciences, Debre Berhan University, Debre Berhan, Ethiopia

**Keywords:** husbands’ knowledge, obstetrical danger signs, cross-sectional, Raya Kobo district, Ethiopia

## Abstract

**Background:**

The term “danger signs” refers to any symptoms or indicators that suggest a pregnant woman may be at risk during pregnancy. Mothers are often burdened with responsibilities, and the majority of them do not even receive treatment for potential complications, which can ultimately lead to the loss of their lives. This situation highlights the barriers that prevent them from being properly prepared for potential risks. In Ethiopia, various studies have been conducted on male involvement in pregnancy, but none have assessed knowledge of obstetric danger signs (ODS). While a few studies have focused on husbands’ knowledge of obstetric danger signs, there is still variation in the variables examined.

**Objectives:**

The objective of the study was to assess the level of knowledge about obstetrical danger signs and the associated factors among husbands whose wives were pregnant and gave birth within the last 12 months in the Raya Kobo district, North Wollo, Amara Ethiopia, in 2023.

**Methods:**

A community-based cross-sectional study was conducted from 29 April to 30 May 2023 in the Raya Kobo district. A multi-stage stratified random sampling technique was used to select 626 samples. The data were analyzed using SPSS version 26.00 statistical software. Independent variables with a *p*-value of ≤0.25 in the bivariate analysis were considered candidates for multivariate analysis, and a *p*-value of ≤0.05 was considered statistically significant.

**Results:**

Regarding knowledge about obstetric danger signs, 46.3% (95% CI: (42.6 to 50.3%)) of the respondents had good knowledge. The variables significantly associated with knowledge about obstetric danger signs included husbands who attended secondary school (adjusted odds ratio (AOR) = 4.77, 95% CI, (1.42–16.04)), those living in urban areas (AOR = 3.00, 95%CI = (1.59, 7.57)), those with an average monthly income between 3,001 and 5,000 birr (AOR = 3.35, 95% CI = (1.58, 7.12)), and those with more than five children (AOR = 0.15, 95% CI = (0.05–0.46)).

**Conclusion and recommendations:**

The knowledge of obstetric danger signs among husbands in the Raya Kobo district was limited. The educational status of the husband, average family income, residence, and number of children were significantly associated with the husbands’ knowledge of obstetric danger signs. Therefore, these findings highlight the importance of addressing knowledge gaps through targeted educational programs aimed at improving awareness of obstetric danger signs.

## Introduction

The term “danger signs” refers to any symptoms or indicators that suggest a pregnant woman may be at risk during pregnancy. They are referred to as obstetric danger signs (ODS) and include symptoms such as loss of consciousness, persistent vomiting, severe persistent abdomen pain, vaginal bleeding, swelling of the face, fingers, and feet, blurred vision, severe recurring frontal headache, and high-grade fever (1, 2).

Every pregnant woman is at risk of experiencing unanticipated, sudden problems related to ODS (1, 3). Pregnancy and its related complications remain one of the major causes of maternal morbidity and mortality worldwide, with over half a million women dying each year due to pregnancy-related causes. Almost all of these (87%) maternal deaths occur in low-income countries (3, 4). Direct obstetric factors, such as severe bleeding, pregnancy-induced hypertension, unsafe abortion, labor and delivery difficulties, and maternal infections, account for approximately 73% of all maternal deaths (3).

Developing countries experience a maternal mortality rate that is 15 times higher than that of developed countries, and the global burden of maternal mortality is concentrated in these regions, with sub-Saharan Africa accounting for 56% of all reported maternal deaths (5). Maternal mortality remains a significant public health concern in low- and middle-income countries, particularly in sub-Saharan Africa, where Ethiopia is located. One of the most effective strategies for reducing maternal mortality is the early identification and management of obstetric danger signs, which are critical indicators of potential complications during pregnancy, childbirth, and the postpartum period (6). Timely recognition of obstetric danger signs allows for prompt intervention, preventing life-threatening complications such as preeclampsia, obstructed labor, and hemorrhage (7).

The Sustainable Development Goal 3 (SDG 3) aims to reduce the global maternal mortality ratio to less than 70 per 100,000 live births by 2030. In addition to this target, each country has the responsibility and commitment to achieve a target of no more than 140 maternal deaths per 100,000 live births (8, 9). Although the maternal mortality ratio is decreasing globally, its reduction in sub-Saharan countries is occurring at a much slower rate. According to the 2019 Ethiopia Demographic Health Survey (EDHS) mini-report, the maternal mortality and morbidity ratio in Ethiopia was 402 per 100,000 live births. Of these deaths, 30% were related to obstetrical danger signs during pregnancy (4).

Maternal mortality remains a critical health challenge in Ethiopia, particularly in rural areas where access to healthcare services is limited. One of the key factors contributing to the high maternal mortality rate is the delay in recognizing and responding to obstetric danger signs. These signs serve as early warning of potential complications during pregnancy, childbirth, and the postpartum period (10).

In the majority of developing countries, including Ethiopia, the underlying cause of maternal death during pregnancy is attributed to three crucial delays, even when ODS occur: delay in identifying danger signs and making the decision to visit the health facility, delay in reaching the health facility, and delay in receiving appropriate and adequate care at the health facility (1, 2).

The majority of women in Ethiopia have a low social status, and the decision-making power regarding access to and use of care is dominated by men. This factor can significantly contribute to adverse pregnancy outcomes (11, 12). A study conducted by Mbizvo and Say analyzed the factors that reduced maternal mortality in Bangladesh, Morocco, and Rwanda. The findings revealed that maternal mortality rates decreased by 20–50% through increased skilled birth attendance and efforts to educate husbands about obstetric danger signs (30).

Several studies conducted in various regions of Africa (including Nigeria, Tanzania, Kenya, and Ethiopia) showed that male partners had a low level of knowledge about obstetric danger signs during pregnancy and childbirth. Only 22.4 to 56.2% of them were able to identify at least two or more of the listed danger signs (2, 13–15).

When husbands fail to recognize the danger signs during pregnancy, it can affect the health of both the mother and the fetus, leading to conditions such as anemia, infections, prematurely ruptured membranes, neonatal morbidity and mortality, abortion, antepartum hemorrhage, and illness or death of the mothers (5, 16). Moreover, a lack of knowledge regarding obstetric danger signs is associated with a delay in the utilization of skilled care, which contributes to an increase in maternal mortality and morbidity (17).

Husbands, particularly in rural Ethiopia, play a key role in influencing the first two delays. As primary decision-makers in many households, they are often responsible for determining when and whether their wives should seek medical care during pregnancy and childbirth (18). If husbands lack knowledge about obstetric danger signs, such as severe vaginal bleeding, convulsions, and prolonged labor, it can lead to significant delays in recognizing complications and deciding to seek timely care, thereby contributing to poor maternal and neonatal outcomes (10).

The World Health Organization (WHO) advocates for the inclusion of men in maternal health strategies as their participation can lead to better care-seeking behaviors, timely interventions, and improved overall maternal well-being (19). Limited awareness of obstetric danger signs among husbands contributes to delays in seeking care, which is a known factor in increasing the risk of maternal mortality in rural Ethiopia. However, maternal deaths are influenced by multiple factors (10, 19).

In Ethiopian cultural practices, men are at the top of the decision-making hierarchy, including decisions regarding the healthcare of their spouses. Therefore, they become a major factor in determining where, when, why, and how many pregnant women can access antenatal care, delivery services, and postpartum care. Husbands’ knowledge of obstetric danger signs, such as severe headache, severe bleeding, convulsions, and prolonged labor, can directly impact the timeliness of seeking care. Husbands with insufficient knowledge of obstetric danger signs may fail to recognize when their wives are in need of urgent medical attention, leading to delays that could result in adverse maternal or neonatal outcomes.

Various studies have been conducted in Ethiopia on male involvement during pregnancy. Despite the acknowledged importance of male involvement in maternal health, there is limited research on husbands’ knowledge of obstetric danger signs. In addition, there is still considerable variation in the variables studied, and no research has been conducted specifically in rural districts such as Raya Kobo.

This study aimed to assess husbands’ knowledge of obstetric danger signs in the Raya Kobo district and to identify the factors associated with this knowledge. Understanding these factors can guide public health interventions aimed at improving male engagement in maternal health and help design targeted educational programs to increase husbands’ awareness of obstetric danger signs, ultimately reducing delays in care-seeking and improving maternal outcomes.

Therefore, incorporating variables, such as distance from nearby health facilities, and sources of information would help assess husbands’ knowledge of obstetric danger signs and the associated factors in the Raya Kobo district.

## Methodology

### Study area

This study was conducted in the Raya Kobo district, North Wollo Zone, Amara Regional state, Ethiopia, which is 570 Km away from Addis Ababa, the capital city of Ethiopia, and 409 km from Bahir Dar, the capital of the regional state. According to the 2007 Census conducted by the Central Statistical Agency (CSA) of Ethiopia, the district had a total population of 221,958. There were 2,659 pregnant women in the district. The district consists of 37 rural and five urban kebeles. It has 1 hospital, 7 health centers, and 42 health posts. Each health post has two health extension workers (HEWs) (20).

### Study design and period

A community-based cross-sectional study was conducted from 29th April to 30th May 2023.

### Participants

The source population included all husbands living in the Raya Kobo district whose wives were pregnant or had given birth within the 12 months prior to the study period. The study population consisted of all husbands living in the selected 11 kebeles whose wives were pregnant or had given birth within the 12 months prior to the study period.

### Eligibility criteria

Inclusion criteria: all husbands living in the Raya Kobo district for 6 months and above were included in this study.

Exclusion criteria: husbands who were critically ill and unable to participate in the interview during the data collection period were excluded from the study.

### Sample size determination

The sample size was determined using a single population proportion formula based on the levels of husbands’ knowledge (*p* = 44%) from a previous study in the Amara region’s Andede district (35). The sample size was calculated as follows:

$\begin{array}{c} vn1=\left(Za/2\right)2p.q\;d2=z\;2x\left(1-p\right)\;d2\\ =\left(\left(1.96\right)2^{*}\left(0.44^{*}0.56\right)\right)/\left(0.05\right)2=379 \end{array}$

Considering a 1.5 design effect and a 10% non-response rate, the total sample size was calculated to be 626.

The second largest sample size was calculated using EP Info V.7, with a maximum result of 189. Therefore, the overall final sample size was determined by considering the first objective, which was 379, a 1.5 design effect, and a 10% non-response rate, resulting in a total sample size of 626.

### Sampling technique and procedure

**Figure 1:**
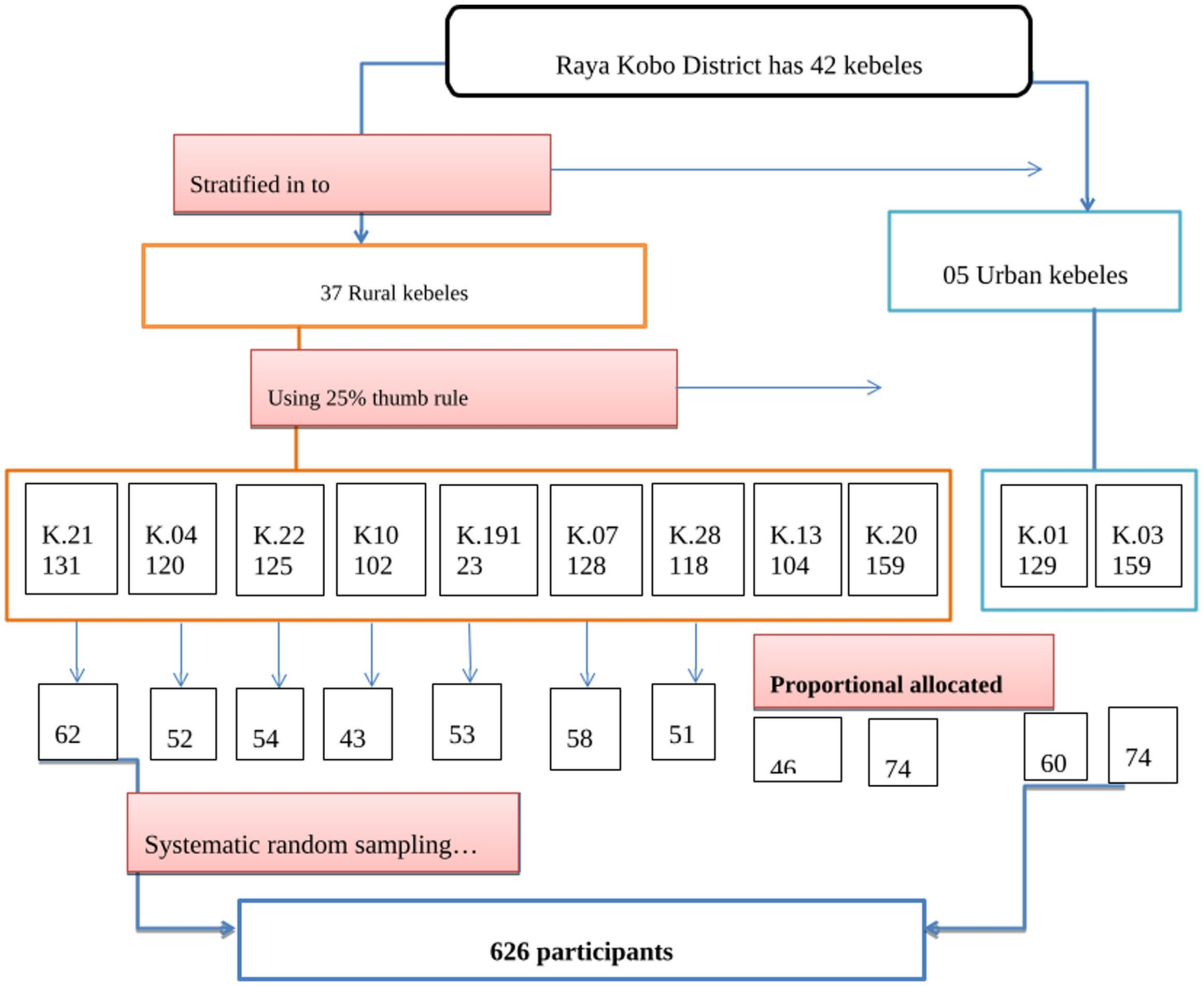
Schematic presentation of the sampling procedure.

A multi-stage stratified random sampling technique was used to select participants. The district was divided into urban and rural areas, with nine kebeles selected from the rural area and two kebeles from the urban area using random sampling and considering a minimum sample size of 25%. Data on husbands whose wives were pregnant or had given birth within the past 12 months were collected through a survey before the actual data collection. Husbands of mothers who were single, divorced, or widowed in the selected kebeles were excluded from the study. After determining the number of participants, the sample size for each kebele was proportionally allocated, and eligible women were selected using a systematic sampling technique. The interval was determined by dividing the total number of eligible women in the study area, which was estimated to be 1,337, by the sample size of 626, resulting in an interval of 2 (*K*=N/n, 1,337/626 = 2). The eligible women were initially selected using the lottery method from a list of folders, and after selecting these women, the researchers then proceeded to approach their husbands to administer the questionnaire (Figure 1).

### Data collection method

Data were collected using face-to-face interview techniques and structured questionnaires adapted from various studies (19, 21). The information collected included sociodemographic and economic characteristics (maternal age, husband’s age, residence, income, educational status of the woman/husband, and occupational status), infrastructure (availability of health facilities and distance from a health facility), obstetrical characteristics (gravidity, parity, and place of delivery), and husbands’ awareness of danger signs of obstetric complications at three stages. A total of eight female HEWs and three BSc midwifery graduates were hired as data collectors and were supervised by two MPH students.

Dependent variable: the dependent variable was the husbands’ knowledge of obstetric danger signs.

Independent variables: the independent variables included sociodemographic and socioeconomic characteristics (maternal age, husband’s age, residence, educational status of the woman/husband, occupational status of the woman/husband, and family income), obstetrical characteristics (gravidity, parity, place of delivery, previous history of danger signs, and male involvement in antenatal care (ANC) follow-up), infrastructure (availability of health facilities and distance from a health facility), and sources of information (radio, TV, decision-making by the husband, and health professionals).

Husbands’ good knowledge of obstetrical danger signs: this refers to men who responded to 26 questions about obstetric danger signs during any of the three phases (pregnancy, childbirth, or the post-partum period) and scored above or equal to the median score (20, 22).

### Data processing and analysis

Each completed questionnaire was checked manually for completeness before data entry. The data were coded and entered into EpiData (V.4.6), cleaned to check for accuracy and consistency, and any errors identified were corrected. The final data were exported to SPSS (IBM-26) for further cleaning and analysis. Logistic regression was performed, and the variables with a 95% CI and a *p*-value of <0.05 were considered significant. The strength of the statistical association was determined by the adjusted odds ratio (AOR) and 95% CI. The assumptions of multiple logistic regression were fulfilled, and the goodness of fit was tested using the Hosmer–Lemeshow statistical test (0.12). Multicollinearity was assessed based on the maximum number of variables. The important findings’ frequencies, proportions, means, and standard deviations were summarized and presented using tables, figures, and narratives.

### Data quality assurance

To maintain the data quality, a reliability test was performed using Cronbach’s *α*, which yielded a value of 0.787. A validity test was conducted by a senior expert, and the Pearson correlation coefficient was calculated using SPSS software. Data collectors received training to further uphold the quality of data. A pretest was conducted on 5% of the study participants from a similar population in the Guba Lafto district, located outside the study area. Based on the pretest feedback, some questions were rephrased and inappropriate skipping patterns were corrected. The questionnaires were initially prepared in English, then translated into Amharic (the national language), and subsequently translated back into English by two different language experts to ensure consistency and clarity.

## Results

### Sociodemographic and socioeconomic characteristics of the husbands

Data collection was planned for 626 participants, and 620 completed the interviews, resulting in a resonse rate of 99.04%. The mean age of the respondents was 34.68 (SD ± 6.46) years. A total of 270 (43.5%) respondents were between the ages of 26 and 35 years. Among the study participants, 209 (33.7%) had primary education, while only 55 (8.9%) had attended college or higher education. A total of 408 (65.8%) respondents lived in rural areas (Table 1).

**Table 1:** Distribution of the respondents and their wives by socio-demographic and economic characteristics in the Raya Kobo district, April 2023 (*n* = 620).

Variable	Characteristic	Frequency	Percentage (%)
Age of the respondent	25 and below	66	10.7
	26–35	270	43.5
	36–45	217	35.0
	46 and above	67	10.8
Age of the wife	24 and below	145	23.4
	25–29	303	48.9
	30–34	92	14.8
	35 and above	80	12.9
Educational status of the husband	Unable to read and write	45	7.3
	Able to read and write	159	25.6
	Primary school	209	33.7
	Secondary school	152	24.5
	College and above	55	8.9
Educational status of the wife	Unable to read and write	86	13.8
	Able to read and write	147	23.7
	Primary school	267	43.1
	Secondary school	81	13.1
	Collage and above	39	6.3
Occupational status of the husband	Farmer	346	55.8
	Governmental employee	155	25.0
	Merchant	60	9.7
	Daily labor	59	9.5
Occupational status of the wife	Housewife	412	66.5
	Governmental employee	93	15.0
	Merchant	107	17.2
	Daily labor	8	1.3
Average family income	1,500 birr and below	145	23.4
	1,501–3,000 birr	268	43.2
	3,001–500 birr	102	16.5
	5,001 birr and above	105	16.9
Residence	Urban	212	34.2
	Rural	408	65.8

### Obstetric characteristics of the respondents

The obstetric characteristics of the respondents were determined based on the condition of their wives, as reported by the husbands. Among the respondents’ wives, 492 (79.4%) were pregnant during the data collection. Regarding the number of pregnancies, 511 (82.4%) of the respondents’ wives were multigravida, and 376 (60.6%) husbands helped their wives comply with the instructions received at health institutions during ANC visits. The majority of the respondents, 600(98.6%), reported that there was a health facility available in their kebeles, but only 99 (16.0%) of them lived within 30 min of a health facility. Similarly, 108 (17.4%) of the respondents needed to walk distances greater than 90 min (Table 2).

**Table 2:** Distribution of the respondents and their wives by obstetric characteristics in the Raya Kobo district, April 2023.

Variable	Characteristics	Frequency	Percentage (%)
Current status of their wives (*n* = 620)	Pregnant	492	79.4
	Breastfeeding	128	20.6
Husbands’ involvement during the ANC visit (*n* = 620)	Yes	376	60.6
	No	244	39.4
Number of gravidity (*n* = 620)	Primigravida	109	17.6
	Multigravida	511	82.4
Place of birth of the last delivery (*n* = 449)	Home	91	20.27
	Health facility	358	79.73
History of obstetric complication (*n* = 511)	Yes	155	30.33
	No	356	69.67
Number of children (*n* = 620)	2 and below	282	45.5
	3 and 4	227	36.6
	5 and above	111	17.9

### Infrastructure-related factors

Of the respondents, 600 (96.8%) reported that health facilities were available in their kebeles, and only 216 (36%) of the respondents reported that it took them less than 30 min to reach the nearest health facility (Table 3).

**Table 3:** Availability of a health facility and the time it takes to reach it in the Raya Kobo district, 2023.

Variable	Character	Frequency	Percentage
Availability of a health facility (620)	Yes	600	96.8%
	No	20	3.2%
The time it takes to reach the nearest health facility (600)	30 min and below	216	36%
	>30 min above	384	64%

### Sources of information

**Figure 2:**
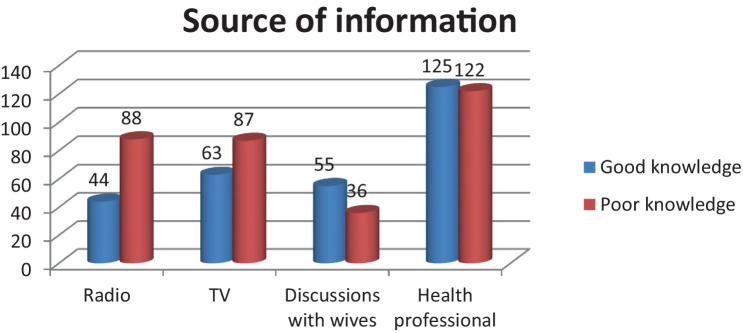
Source of information for the husband on ODS in Raya Kobo district, April (2023).

The findings of this study indicated that the majority of the participants, 247(39.8%), who gathered information about obstetric danger signs received it from health professionals (Figure 2).

### Knowledge of obstetric danger signs

**Figure 3:**
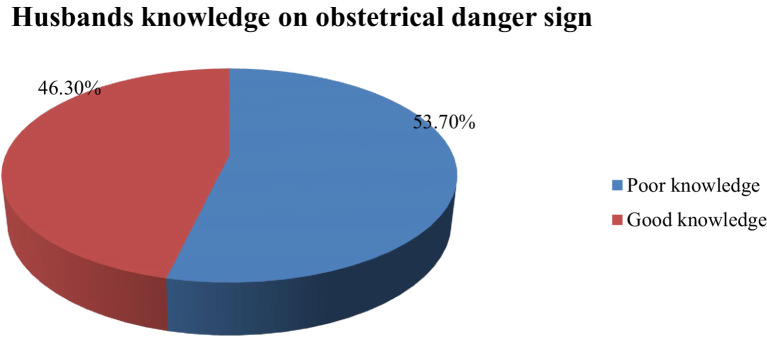
Knowledge of obstetric danger signs among husbands in the Raya Kobo District April 2023.

The knowledge of obstetric danger signs among the respondents was calculated for each period using the median score cut-off point of 34. It was categorized as “good knowledge” when the average score was above the median score, and it was categorized as “poor knowledge” when the average score was below the median score. Of all respondents, 46.3% (95% CI: 42.6–50.3) had good knowledge (Figure 3).

### Knowledge of obstetric danger signs during pregnancy, delivery, and postpartum periods

When computing the phase one variable to identify those above the median score, 261 (42.1%) of the respondents, with a 95% CI of (38.1, 46.0), had good knowledge during the postpartum period. In this phase, severe vaginal bleeding after delivery was reported to be a high-scoring obstetric danger sign, mentioned by 348 (56.1%), while severe weakness was reported to be a lower-scoring obstetric danger sign, mentioned by 185 (29.8%). In another computed variable, 36.6% of the respondents had good knowledge throughout pregnancy. A total of 422(68.1%) respondents mentioned membrane rupture before labor and 411 (66.3%) respondents mentioned severe vaginal bleeding as high-scoring obstetric danger signs, while 92 (14.8%) mentioned difficulty breathing as a lower-scoring obstetric danger sign. Lastly, during delivery, 26% of the husbands mentioned in phase two had a median score. A total of 301 (48.1%) identified placenta retention (placenta not detaching before 30 min) as the highest-scoring obstetric danger sign, while 106 (17.1%) mentioned high-grade fever as a lower-scoring danger sign (Table 4).

**Table 4:** Knowledge of obstetric danger signs during pregnancy, delivery, and postpartum periods among the husbands in the Raya Kobo district, April 2023.

Variable	Frequency (Yes)	Percentage (%)
Part 1: during pregnancy		
Severe vaginal bleeding	411	66.3%
Severe headache	280	45.2%
Blurring of vision	216	34.8%
Convulsion	204	32.9%
Swelling of hand and face	153	24.7%
High-grade fever	101	16.3%
Difficulty breathing	92	14.8%
Severe weakness	100	16.1%
Severe abdominal pain	243	39.2%
Reduced fetal movement	243	39.2%
Membrane rupture	422	68.1%
Part 2: during delivery		
Vaginal bleeding	205	33.1%
Severe headache	129	20.8%
Convulsion	111	17.9%
High-grade fever	106	17.1%
Labor >12 h	266	42.9%
Placenta not detached after 30 min	301	48.5%
Part 3: during postpartum		
Severe vaginal bleeding	348	56.1%
Severe headache	226	36.5%
High-grade fever	187	30.2%
Offensive vaginal discharge	229	36.9%
Convulsion	203	32.7%
Severe weakness	185	29.8%
Blurring of vision	197	31.8%
Swelling of hand and face	199	32.1%
Difficulty breathing	236	38.1%

### Factors associated with the husbands’ knowledge of obstetrical danger signs

To identify the factors associated with the husbands’ knowledge of obstetric danger signs, binary and multivariable logistic regression analyses were conducted with the dependent and independent variables. In the binary logistic regression, the age of the husband, the occupation of the husband, the level of education of the husband, family income, residence, the husband’s involvement in ANC visits, the wife’s history of bad obstetric signs, number of children, and the level of education of the wife were significantly associated with the husbands’ knowledge of obstetric danger signs.

The multivariable analysis was performed to identify independent predictors of the husbands’ knowledge regarding obstetrics danger signs. After controlling for possible confounders, the husband’s education, residence, number of children, and family income were identified as the independent predictors of the husbands’ knowledge of obstetrics danger signs in the Raya Kobo district. These results also showed that the husbands who had secondary school education were 4.8 times (AOR = 4.77, 95% CI, (1.42–16.04)) more likely to have good knowledge of obstetrics danger signs than those who were unable to read and write.

The husbands in families with more than five children were 85% (AOR = 0.15: 95% CI; (0.05–0.46)) less likely to have good knowledge of obstetric danger signs compared to those with fewer than two children. The husbands with an average monthly income of 3,001–5,000 birr were 3.4 times (AOR = 3.35, 95% CI = (1.58, 7.12)) more likely to have good knowledge compared to those with an average monthly income of less than 1,500 birr. The husbands living in urban areas were 3 times (AOR = 3.00, 95%CI = (1.59, 7.57)) more likely to have good knowledge than those living in rural areas (Table 5).

**Table 5:** Multivariate and bivariate logistic regression analyses showing factors independently associated with the husbands’ knowledge of obstetrics danger signs in the Raya Kobo district, 2023.

Characteristic with Variable		Knowledge		COR (95% CI)	AOR (95% CI)
		Good knowledge	Poor knowledge		
Age of the respondent	25 and below	34 (51.5)%	32 (48.5)%	1	1
	26–35	141 (52.2)%	129 (47.8)%	1.03 (0.60–1.67)	0.82 (0.35–1.95)
	36–45	93 (42.9)%	124 (57.1)%	0.71 (0.41–1.23)	1.20 (0.44–3.29)
	46 and above	19 (29.4)%	48 (71.6)%	0.34 (0.18–0.76)	1.96 (0.51–7.54)
Husbands’ education	Unable to read and write	15 (33.4%)	30 (66.6%)	1	1
	Able to read and write	43 (27%)	116 (73%)	0.74 (0.36–1.51)	1.43 (0.55–3.71)
	Primary school	74 (35.4%)	135 (64.6%)	1.09 (0.55–2.17)	1.79 (0.59–5.42)
	Secondary school	117 (75%)	38 (25%)	**6.16 (3.24–13.8)**	4.77 (1.42–16.04)*
	Collage and above	38 (69.1%)	17 (30.9)	4.47 (1.92–10.39)	1.65 (0.45–6.05)
Husbands’ occupation	Farmer	96 (27.7)%	250 (72.3)%	1	
	Government employee	109 (69.3)%	46 (29.7%)	6.17 (4.06–9.37)	1.57 (0.81 to 3.05)
	Merchant	44 (73.3)%	16 (26.7)%	7.16 (3.86–13.3)	2.16 (0.9 to 5.17)
	Daily labor	38 (64.4)%	21 (35.6)%	4.7 (2.63–8.44)	1.23 (0.54 to 2.8)
Residence	Urban	159 (75)%	53 (25)%	**6.56 (4.51–9.54)**	3.00 (1.59–5.57)**
	Rural	128 (21.4)%	280 (68.6)%	1	1
Husbands’ involvement in the ANC visit	No	85 (34.8%)	159 (65.2%)	1	1
	Yes	202 (53.7%)	174 (46.3%)	2.17 (01.56–3.02)	1.73 (0.81 to 3.68)
History of obstetric danger signs	No	150 (42.4%)	206 (57.6%)	1	1
	Yes	78 (49.4%)	80 (50.6%)	0.78 (0.53–1.13)	1.22 (0.54 to 2.74)
Number of children	2 and below	168 (59.9)%	114 (40.2)%	1	1
	3 and 4	94 (41.4)%	133 (58.6)%	0.48 (0.34–0.68)	0.79 (0.45–1.41)
	5 and above	25 (22.5)%	86 (77.5)%	**0.20 (0.12–0.33)**	0.15 (0.05–0.46)*
Family income	1,500 birr and below	37 (24.5)%	108 (74.5)%	1	1
	1,501–3,000 birr	92 (34.3)%	176 (65.7)%	1.53 (0.97–2.39)	1.15 (0.67–1.99)
	3,001–500 birr	76 (74.5)%	26 (25.5)%	**8.53 (4.77–15.26)**	3.35 (1.58–7.12)*
	5,001 birr and above	82 (78.1)%	23 (21.9)%	10.41 (5.74–18.85)	1.68 (0.66–4.26)

*significant at *p* value  < 0.05, **significant at *p* value  < 0.01.

## Discussion

The findings of this study indicated that the magnitude of the husbands’ knowledge of obstetric danger signs in the overall combination of three phases (prenatal period, childbirth, and postnatal period) was 46.3%, with a 95% CI of 42.6 to 50.3%. This finding is lower than that in Nigeria, where research conducted in two different study areas found that 53.8 and 51.9% of men had good knowledge of obstetric danger signs in pregnancy, as well as 53.3% in Dessie (12, 21, 22). This variation may be due to socio-economical differences between the studies conducted in Nigeria and this study, where the majority of the participants lived in rural areas that had less access to sources of information about obstetric danger signs and a lower likelihood of wives attending ANC follow-up visits, which would provide opportunities for sharing knowledge on danger signs. However, this finding was higher than the findings of studies conducted in the Musoma district, Mara region, Tanzania (32%) and southern Ethiopia (42.2%) (23).

Possible reasons for the higher prevalence and the differences observed might include healthcare system disparities between the countries, especially the utilization of community mobilization on maternal health in Ethiopia by health extension workers. Additionally, the time gap between studies may have contributed, as wives’ utilization of maternal care may have improved, which in turn could have led to an increase in husbands’ knowledge of danger signs. The study found that the husbands who had completed secondary school were 4.8 times (AOR = 4.77, 95% CI, (1.42–16.04)) more likely to have good knowledge of obstetric danger signs than those who were unable to read and write. This finding is in line with a study conducted in Southwest Nigeria (24). This may be due to the fact that education is an important tool for increasing understanding and access to information about obstetric danger signs and their outcomes through various electronic media. In addition, closer collaboration between schools and local health services may enhance the delivery of reproductive health education. The place of residence was significantly associated with the husbands’ knowledge of obstetric danger signs. The husbands living in urban areas were 3 times (AOR = 3.00, 95%CI = (1.59, 7.57)) more likely to have good knowledge than those living in rural areas. This finding is similar to a study conducted in the Andede district (25). This could be because the respondents living in urban areas in the present study had greater access to information compared to those in rural areas, as most messages are transmitted through various media available in urban settings. However, this finding is in contradiction with a study conducted in the Chencha district, Gamo Gofa Zone, southern Ethiopia, which showed that husbands living in rural areas were 8.4 times more knowledgeable than those living in urban areas (11). This discrepancy may be due to the fact that the husbands living in rural areas in this study had limited sources of information and lower educational backgrounds compared to those living in urban areas.

The number of children in this study was a significant factor associated with the husbands’ awareness. The husbands in families with more than five children were 85% times (AOR = 0.15; 95% CI: 0.05–0.46) less likely to have good knowledge of obstetric danger signs compared to those with fewer than two children. The finding is consistent with a study conducted in Tanzania (26).A possible reason for this is that husbands with many children tend to prioritize income generation, leading to longer working hours and multiple sources of employment (27). This situation leaves them with limited time to attend antenatal care (ANC) visits or to recognize and respond to obstetric danger signs, such as prolonged labor, severe bleeding, or reduced fetal movement, all of which require immediate attention. However, a study conducted in Nigeria showed no significant difference in the knowledge of danger signs regarding the number of children in the family (28). The reason for this discrepancy may be the large sample size used as larger sample sizes increase the probability of detecting associations between variables.

The total average monthly income of the family was also a factor associated with the husbands’ knowledge of obstetric complications. The husbands with an average monthly income of 3,001–5,000 birr were 3.4 times (AOR = 3.35, 95% CI = (1.58, 7.12)) more likely to have good knowledge compared to those with an average monthly income of less than 1,500 birr. This finding is in line with studies conducted in South Ethiopia and Uganda, where economic status was also significantly associated with men’s awareness of danger signs of obstetric complications (28, 29). This may be because men with higher economic status are more likely to be exposed to health education provided by health institutions. In addition, they can afford radios, TVs, and other media to access more information about maternal health. Higher-income households tend to exhibit better health-seeking behaviors and greater engagement in maternal health, as they are less constrained by financial limitations. This access to information and services enables them to pay more attention to their wives’ pregnancies, increasing the likelihood of early recognition and intervention in the case of obstetric danger signs.

### Strengths and limitations of this study

The study was community-based, which allowed it to reflect the experiences of the husbands during the study period. It also utilized a large sample size, making the findings more representative. However, a limitation of the study is the potential for recall bias, even though the recall period was reduced to 1 year. The husbands were asked to remember events that occurred up to 1 year before the study, and the data were collected by having them mention the obstetric danger signs they knew and the activities they had undertaken, without providing them with options to choose from.

## Conclusion

The husbands’ knowledge of obstetric danger signs in the Raya Kobo district was limited. Severe vaginal bleeding was the most frequently identified obstetric danger sign during the antepartum, intrapartum, and postpartum periods. The educational status of the husband, average family income, residence, and number of children were significantly associated with the husbands’ knowledge of obstetric danger signs. These findings highlight the importance of addressing knowledge gaps through targeted educational programs aimed at improving awareness of obstetric complications. Enhancing this awareness among husbands could play a crucial role in promoting timely healthcare-seeking behaviors and improving maternal health outcomes in the study area.

## Data Availability

The data sets used or analyzed during the current study are available from the corresponding author upon reasonable request.

## References

[1] WHO, U. and W.B. UNFPA. Trends in maternal mortality: 1990 to 2015. Geneva: World Health Organization (2015).

[2] Woldeamanuel GG Lemma G Zegeye B. Knowledge of obstetric danger signs and its associated factors among pregnant women in Angolela Tera District, Northern Ethiopia. BMC Res Notes. (2019) 12:1–6. doi: 10.1186/s13104-019-4639-8 31547838 PMC6755683

[3] Organization, W.H. Trends in maternal mortality 2000 to 2017: Estimates by WHO, UNICEF, UNFPA, World Bank Group and the United Nations population division. Geneva, Switzerland: Executive summary World Health Organization (2019).

[4] Brun M Monet JP Moreira I Agbigbi Y Lysias J Schaaf M., Implementation manual for developing a national network of maternity units-improving emergency obstetric and newborn care (EmONC), United Nations Population Fund. United Nations Popul Fund. (2020).

[5] Stafford-Smith M Griggs D Gaffney O Ullah F Reyers B Kanie N. Integration: the key to implementing the sustainable development goals. Sustain Sci. (2017) 12:911–9. doi: 10.1007/s11625-016-0383-3, PMID: 30147763 PMC6086249

[6] Organization, W.H. Trends in maternal mortality: 1990 to 2015. Geneva: WHO (2015). 2018 p.

[7] Alem AZ Yeshaw Y Liyew AM Tesema GA Alamneh TS Worku MG. Timely initiation of antenatal care and its associated factors among pregnant women in sub-Saharan Africa: a multicountry analysis of demographic and health surveys. PLoS One. (2022) 17:e0262411. doi: 10.1371/journal.pone.0262411, PMID: 35007296 PMC8746770

[8] Woldegiorgis MA Bhowmik J Wubegzier M. Trends in reproductive health indicators in Ethiopia: 2000–2014. Int J Healthcare. (2017) 3:17–25.

[9] Kurji J. Assessing the determinants of maternal healthcare service utilization and effectiveness of interventions to improve institutional births in Jimma Zone. Ethiopia: Université d'Ottawa/University of Ottawa (2021).

[10] Hailu M Gebremariam A Alemseged F. Knowledge about obstetric danger signs among pregnant women in Aleta Wondo District, Sidama Zone, southern Ethiopia. Ethiop J Health Sci. (2010) 20:25–32. doi: 10.4314/ejhs.v20i1.69428, PMID: 22434957 PMC3275898

[11] Debiso AT Gello BM Malaju MT. Factors associated with men’s awareness of danger signs of obstetric complications and its effect on men’s involvement in birth preparedness practice in southern Ethiopia, 2014. Adv Public Health. (2015) 2015:1–9. doi: 10.1155/2015/386084

[12] Tadesse M Boltena AT Asamoah BO. Husbands’ participation in birth preparedness and complication readiness and associated factors in Wolaita Sodo town, southern Ethiopia. African J Prim Health Care Fam Med. (2018) 10:1–8. 10.4102/phcfm.v10i1.1471 PMC5913778 29781684

[13] Okoror CEM Omuemu VO. Knowledge of obstetric danger signs among antenatal clinic attendees in south–South Nigeria. Health Care Women Int. (2021) 44:1363–78. doi: 10.1080/07399332.2021.1941025 34339345

[14] Winner MBF Do M. Knowledge of obstetric danger signs among adolescent and adult mothers in Tanzania: a secondary data analysis. Women's Reprod Health. (2021) 8:279–93. doi: 10.1080/23293691.2021.1976049

[15] Orwa J Gatimu SM Mantel M Luchters S Mugerwa MA Brownie S. Birth preparedness and complication readiness among women of reproductive age in Kenya and Tanzania: a community-based cross-sectional survey. BMC Pregnancy Childbirth. (2020) 20:1–9. doi: 10.1186/s12884-020-03329-5 PMC7574438 33076869

[16] Arefaynie M Kefale B Yalew M Adane B Dewau R Damtie Y. Number of antenatal care utilization and associated factors among pregnant women in Ethiopia: zero-inflated Poisson regression of 2019 intermediate Ethiopian demography health survey. Reprod Health. (2022) 19:1–10. doi: 10.1186/s12978-022-01347-4 35123503 PMC8817592

[17] Suandi D Williams P Bhattacharya S. Does involving male partners in antenatal care improve healthcare utilisation? Systematic review and meta-analysis of the published literature from low-and middle-income countries. Int Health. (2020) 12:484–98. doi: 10.1093/inthealth/ihz073, PMID: 31613327 PMC11701106

[18] Getachew F Sintayehu A Damitew S Endalkachew F Gebeyaw M. Mens knowledge towards obstetric danger signs and their involvement on birth preparedness in Aneded woreda, Amhara regional state, Northwest Ethiopia. Ethio J Public Health Nutri. (2019) 3:39–45.

[19] Organization, W.H. Trends in maternal mortality: 1990–2015: Estimates from WHO, UNICEF, UNFPA, world bank group and the United Nations population division. Geveva, Switzerland: World Health Organization (2015).

[20] Yimer NB Liben ML. Knowledge on intrapartum danger sign influences place of delivery: the case of Raya Kobo District, northeastern Ethiopia. Int J. (2021) 10:44–50. doi: 10.6000/1929-4247.2021.10.02.1

[21] Chattopadhyay A Govil D. Men and maternal health care utilization in India and in selected less-developed states: evidence from a large-scale survey 2015–16. J Biosoc Sci. (2021) 53:724–44. doi: 10.1017/S0021932020000498 32912342

[22] Shitie A Dilnessa T Ayalew S Tadesse B. Knowledge and factors associated with obstetric danger signs among married men in Dessie town, south Wollo, north-East Ethiopia: a community-based cross-sectional study. BMJ Open. (2022) 12:e063936. doi: 10.1136/bmjopen-2022-063936, PMID: 36581977 PMC9438201

[23] Rahman AE Perkins J Islam S Siddique AB Moinuddin M Anwar MR. Knowledge and involvement of husbands in maternal and newborn health in rural Bangladesh. BMC Pregnancy Childbirth. (2018) 18:1–12. doi: 10.1186/s12884-018-1882-2 29914410 PMC6007056

[24] Sekoni O Owoaje E. Male knowledge of danger signs of obstetric complications in an urban city in south West Nigeria. Annals Ibadan Postgraduate Med. (2014) 12:89–95. PMID: 25960699 PMC4415391

[25] Getachew F Abate S Solomon D Fekade E Molla G. Mens knowledge towards obstetric danger signs and their involvement on birth preparedness in Aneded woreda, Amhara regional state, Northwest Ethiopia. Ethiop J Public Health Nutri. (2020) 3:39–45.

[26] August F Pembe AB Mpembeni R Axemo P Darj E. Men’s knowledge of obstetric danger signs, birth preparedness and complication readiness in rural Tanzania. PLoS One. (2015) 10:e0125978. doi: 10.1371/journal.pone.0125978, PMID: 25950814 PMC4423869

[27] Jibro Abdullahi Y., Income diversification and its determinants among rural farm households: the Case of Haramaya district, East Hararghe Zone Oforomia, Ethiopia. 2021, Haramaya university. ir.haramaya.edu.et (2021).

[28] Oguntunde O Nyenwa J Yusuf FM Dauda DS Salihu A Sinai I. Factors associated with knowledge of obstetric danger signs and perceptions of the need for obstetric care among married men in northern Nigeria: a cross-sectional survey. BMC Pregnancy Childbirth. (2019) 19:1–7. doi: 10.1186/s12884-019-2271-1 30971216 PMC6458632

[29] Kakaire O Kaye DK Osinde MO. Male involvement in birth preparedness and complication readiness for emergency obstetric referrals in rural Uganda. Reprod Health. (2011) 8:1–7. doi: 10.1186/1742-4755-8-12 21548976 PMC3118172

[30] Mbizvo MT Say L. Global progress and potentially effective policy responses to reduce maternal mortality. Int J Gynecol Obstet. (2012) 119:S9–S12. doi: 10.1016/j.ijgo.2012.03.009, PMID: 22883916

